# Backbone NMR assignments of HypF-N under conditions generating toxic and non-toxic oligomers

**DOI:** 10.1007/s12104-018-9822-7

**Published:** 2018-05-21

**Authors:** Jayneil R. Patel, Yingqi Xu, Claudia Capitini, Fabrizio Chiti, Alfonso De Simone

**Affiliations:** 1 Department of Life Sciences, Imperial College London, South Kensington, London, SW72AZ UK; 2 Section of Biochemistry, Department of Experimental and Clinical Biomedical Sciences, University of Florence, Viale Morgagni 50, 50134 Firenze, Italy

**Keywords:** Biomolecular NMR, Protein assignment, HypF-N, Amyloids, Protein oligomers

## Abstract

The HypF protein is involved in the maturation and regulation of hydrogenases. The N-terminal domain of HypF (HypF-N) has served as a key model system to study the pathways of protein amyloid formation and the nature of the toxicity of pre-fibrilar protein oligomers. This domain can aggregate into two forms of oligomers having significantly different toxic effects when added to neuronal cultures. Here, NMR assignments of HypF-N backbone resonances are presented in its native state and under the conditions favouring the formation of toxic and non-toxic oligomers. The analyses of chemical shifts provide insights into the protein conformational state and the possible pathways leading to the formation of different types of oligomers.

## Introduction

HypF plays a chaperone role in the biogenesis of nickel–iron [NiFe] insertion in hydrogenase enzymes from *E. coli* (Maier and Böck 1996). The structure of HypF, lacking its N-terminal acylphosphatase domain, has been previously elucidated by X-ray crystallography (Petkun et al. 2011) (PDB code: 3TSP, 3TSQ, 3TSU, 3TTD, 3TTF). The N-terminal domain of HypF (HypF-N) is a small 11 kDa α/β protein of 91 residues (Rosano et al. 2002) that is structured in a β-sheet of five strands (S1–S5) and 2 α-helices (H1 and H2) in a βαββαβ topology (PDB code: 1GXT, 1GXU) (Rosano et al. 2002). While HypF-N is not associated to any disease, it has been extensively used to elucidate the underlying biomolecular processes of neurodegenerative disorders (Chiti et al. 2001; Campioni et al. 2010; Zampagni et al. 2011). It is indeed well established that the aggregation of otherwise soluble proteins into amyloid fibrils is associated with a number of neurodegenerative conditions, including Alzheimer’s and Parkinson’s diseases, and non-neuropathic conditions such as diabetes type II (Chiti and Dobson 2017). It is now generally acknowledged that the most pernicious species along the pathways of amyloid formation are the small diffusible pre-fibrilar oligomers (Campioni et al. 2010; Fusco et al. 2017). In this context, HypF-N has unique features to enable the elucidation of the molecular basis of the toxicity of protein oligomers, as it can form two different types of protein oligomers that, while being similar in composition, shape, size and morphology, have significantly different toxic effects when incubated with cellular cultures, with only one showing toxic effects. Such a biological diversity has been attributed to the selective interaction with the cellular membrane, with only the toxic oligomers being able to penetrate the membrane and cause an influx of Ca^2+^ ions (Cecchi et al. 2005; Canale et al. 2006; Campioni et al. 2010; Zampagni et al. 2011). Despite the relevance of this protein in the context of amyloid diseases, the assignments of the NMR resonances of HypF-N under the conditions A and B, which respectively generate toxic and non-toxic oligomers, are not available from the BMRB. In the present work we present a detailed characterisation of the general structural properties of HypF-N in its native state and under the two oligomerising conditions. The analysis of chemical shifts clearly identifies a substantial degree of structural and dynamical differences between the protein monomers investigated under the conditions leading to toxic and non-toxic oligomers. This work will boost further high-resolution characterisations in these two states.

## Methods and materials

^1^H, ^13^C, ^15^N, isotopically labelled HypF-N was expressed and purified as previously reported (Calloni et al. 2008), using an N-terminal His-tag construct that was expressed in the *E. coli* strain M15[PREP4] (Qiagen), grown in M9 minimal media by using ^15^N-enriched ammonium chloride and ^13^C-enriched glucose, and purified using a nickel column (Sigma Aldrich). The HypF-N protein was then cleaved from the nickel resin with thrombin from bovine plasma (Sigma Aldrich) overnight at 4 °C in phosphate buffer. Thrombin cleavage generates a non-native N-terminal sequence (Gly-Ser-Ala instead of Met-Ala). This construct is equivalent to that employed in the study identifying the conditions (A and B) to generate toxic and non-toxic HypF-N oligomers (Campioni et al. 2010). The eluted HypF-N was then buffer exchanged into 5 mM sodium acetate, 2 mM dithiothreitol (DTT) buffer at pH 5.5.

NMR measurements were performed on ^13^C, ^15^N labelled HypF-N samples at a concentration of 200 µM. Three independent assignments of the backbone resonances were performed, including native (5 mM sodium acetate, 2 mM DTT, 50 mM sodium phosphate pH 5.5), toxic condition A (50 mM sodium acetate, 2 mM DTT, 12% 2,2,2-trifluoroethanol (TFE) (v/v), pH 5.5) and non-toxic condition B [330 mM sodium chloride, 20 mM trifluoroacetic acid (TFA), pH 1.7]. Each sample contained 10% (v/v) D_2_O for the NMR lock. For the aggregation prone conditions A and B, NMR measurements were performed in a time frame corresponding to the lag phase for aggregation, which enables minimal monomer depletion from the solution. Under the conditions employed in this work, the lag phases were ~ 16 and ~ 24 h for condition A and B, respectively. Measurements of 3D NMR spectra were therefore set to be carried out within these time ranges, requiring fresh samples to be prepared for each 3D measurement.

Assignment of the backbone resonances under these three conditions was performed by a combination of ^1^H–^15^N HSQC, CBCA(CO)NH, HNCACB, HNCO, HN(CA)CO and HNHA spectra, collectively providing chemical shifts for ^1^Hα, ^13^Cα, ^13^Cβ, ^1^HN, ^13^CO and ^15^N atoms. For the non-toxic condition B, an additional HNcocaNH spectrum (Sun et al. 2005) was recorded to aid sequential assignment. NMR was performed at 25 °C using Bruker AVANCE spectrometers operating at proton frequencies of 600 or 800 MHz, both equipped with a triple resonance cryoprobe. NMR data were processed using NMRPipe (Delaglio et al. 1995) and analysed using CCPNAnalysis (Vranken et al. 2005).

## Results

**Fig. 1:**
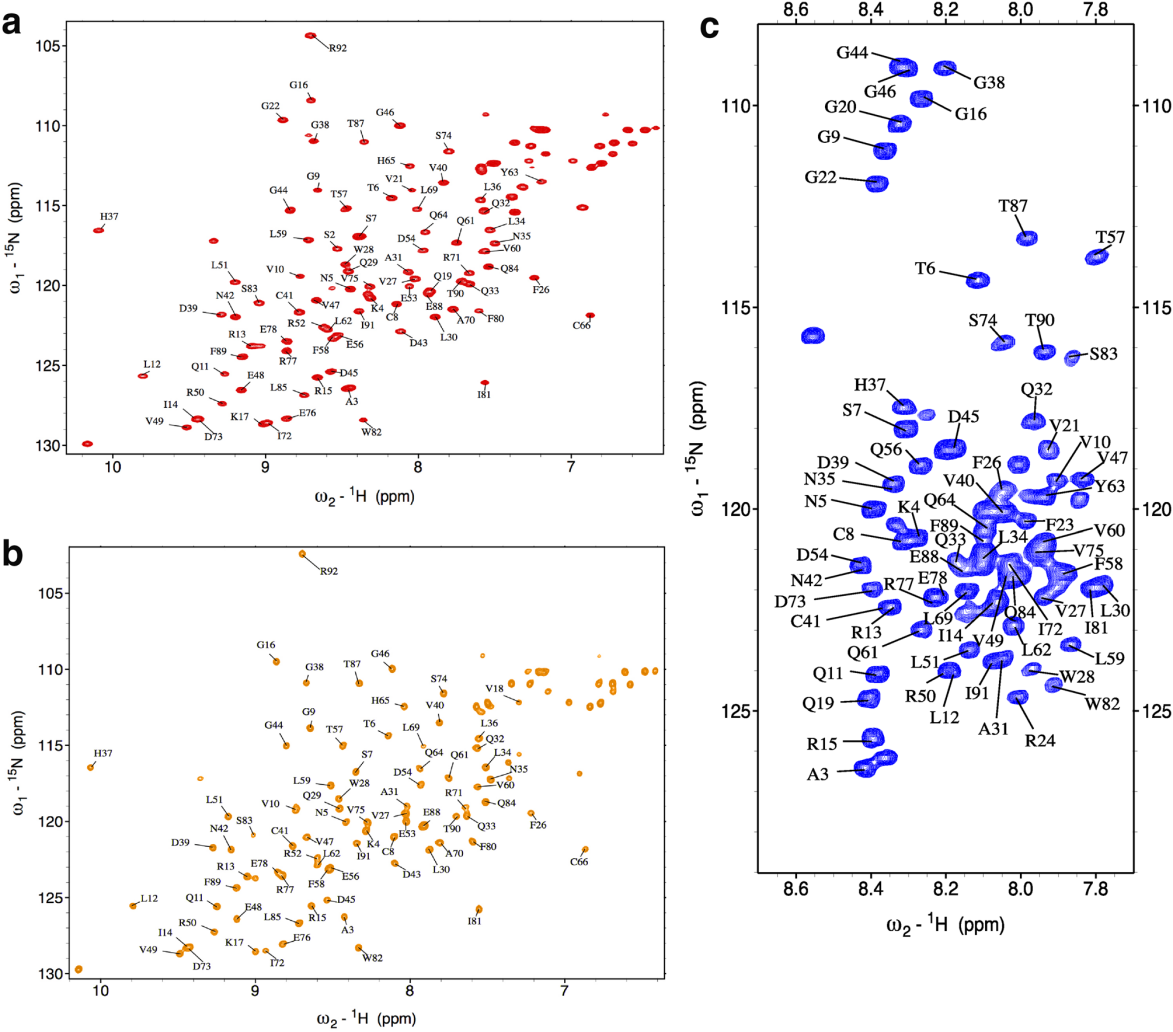
Assigned 2D ^1^H–^15^N HSQC spectra of HypF-N recorded at 25 °C at the ^1^H frequency of 600 MHz. **a** Native state. **b** Condition A. **c** Condition B

**Fig. 2:**
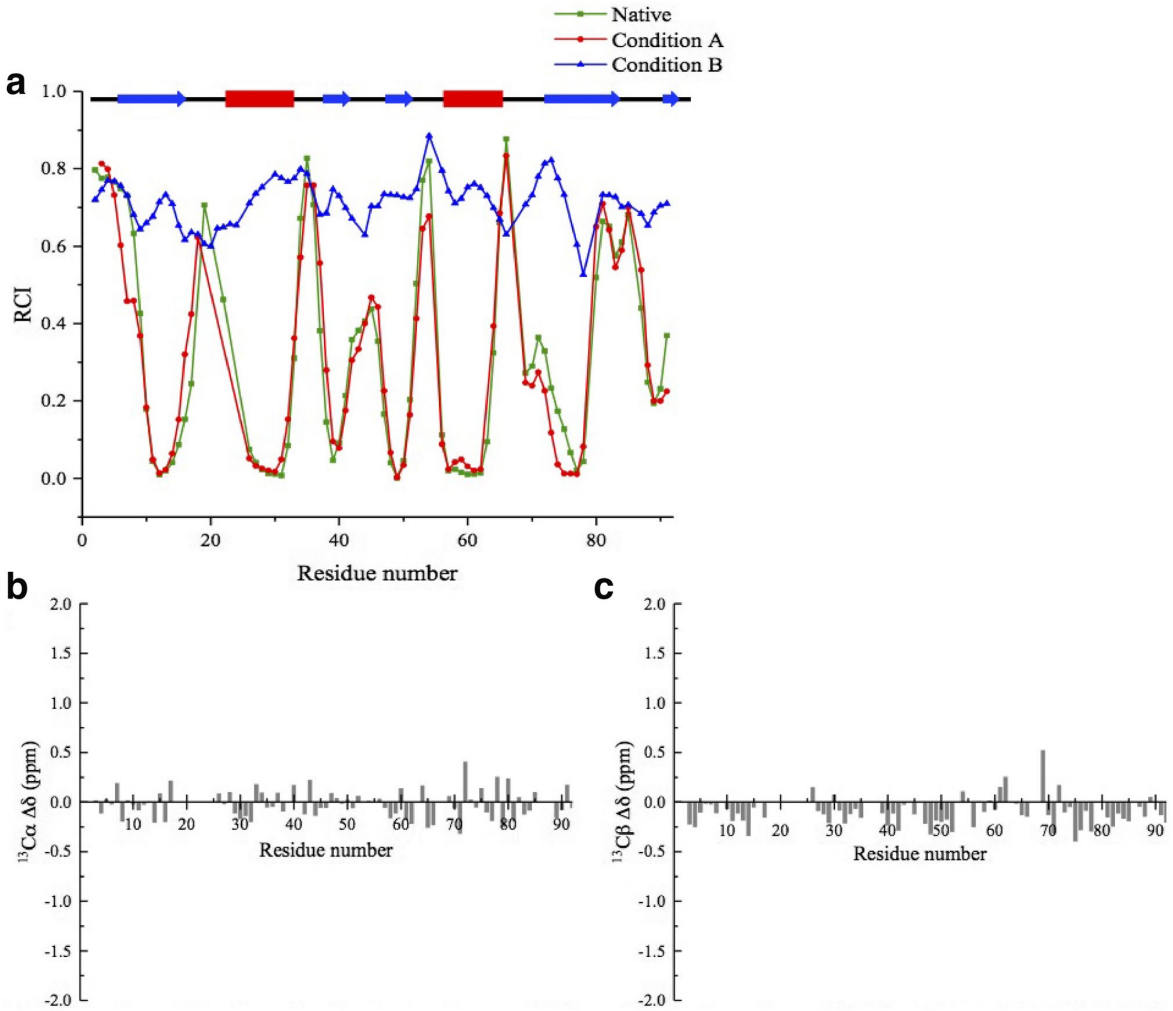
**a** Random coil index estimated using the population of coil regions from δ2D (Camilloni et al. 2012). Native state and aggregation prone states of HypF-N (condition A and condition B) are shown with green, red and blue lines, respectively. Lines are used to connect the data for which RCI values have been calculated based on chemical shifts. The schematic of the native protein secondary structure is shown illustrating the α-helical segments (red blocks) and β-sheet strands (blue arrows). **b**–**c**) Differences between the ^13^Cα (**b**) and ^13^Cβ (**c**) chemical shifts measured for the native state and condition A of HypF-N. Data has been excluded where assignments are missing in one or both conditions

Under native conditions, 81 (out of 86 non-proline) ^1^H–^15^N correlations were assigned across the protein sequence (Fig. 1a). The peak dispersion in the ^1^H–^15^N-HSQC spectrum indicates that HypF-N is well-folded in its native state, as also confirmed by the RCI analysis made using δ2D (Camilloni et al. 2012) (Fig. 2a). Under condition A, the spectral properties suggest that HypF-N is structured in a native-like conformational state (Fig. 1a-b), with ^1^H ^15^N resonances close to those of the native state, except for the catalytic phosphate binding site (residues 18–24), for which no NMR resonances are detected under these conditions. Indeed, the lack of phosphate ions in the condition A likely generates a local conformational exchange that enhances the relaxation of the resonances of the loop 18–24 leading to significant peak broadening, observed in homologous acylphosphotase enzymes (Fusco et al. 2012; De Simone et al. 2011). The similarity of spectral properties between native state and condition A is also shown by the difference in chemical shifts for ^13^Cα and ^13^Cβ (Fig. 2b, c). In contrast, under condition B, HypF-N shows spectral properties that are indicative of an unfolded protein (Fig. 1c), as previously observed (Calloni et al. 2008). The assignment statistics for conditions A and B are listed in Table 1.

**Table 1:** Number of assigned nuclei

	^1^Hα	^13^Cα	^13^Cβ	^13^CO	^15^N	^1^HN
Native^a^	79	78	81	79	81	81
Condition A	72	77	78	77	77	77
Condition B	62	78	79	78	78	78

^a^Unassigned or missing backbone amide resonances in the ^1^H–^15^N HSQC: Native: 1, 18, 20, 23, 24. Condition A: 1, 2, 19–24, 63. Condition B: 1, 29, 36, 43, 53 76, 80, 92

Using the chemical shift values, random coil index was calculated as the population of coil regions in δ2D (Camilloni et al. 2012) (Fig. 2a). This analysis indicates that, in contrast to the native state and condition A, showing structured and coil regions that are consistent with those identified in the X-ray structure of HypF-N (PDB code: 1GXT; Rosano et al. 2002), condition B exhibits significantly high values of RCI throughout the sequence, which is indicative of a highly flexible and disordered state.

In summary, NMR assignments of HypF-N resonances under different oligomerising conditions and in the native state have provided crucial insights into the conformational and structural properties of the protein in its physiological and aggregation-prone states. The assignments will enable further investigations to reveal the detailed mechanisms leading to the formation of toxic and nontoxic HypF-N oligomers.

The assignments have been deposited to the BMRB under the accession codes: 27139 (native), 27137 (toxic) and 27138 (non-toxic).
